# Age-related exacerbation of hematopoietic organ damage induced by systemic hyper-inflammation in senescence-accelerated mice

**DOI:** 10.1038/s41598-021-02621-4

**Published:** 2021-12-01

**Authors:** Tomonori Harada, Isao Tsuboi, Hirotsugu Hino, Miyuki Yuda, Yoko Hirabayashi, Shuichi Hirai, Shin Aizawa

**Affiliations:** 1 Division of Anatomical Science, Department of Functional Morphology, Nihon University School of Medicine, 30-1 Oyaguchi-kamicho, Itabashi-ku, Tokyo, 173-8610 Japan; 2 Center for Biological Safety and Research, National Institute of Health Sciences, Kawasaki, 210-9501 Japan

**Keywords:** Experimental models of disease, Haematopoietic system, Haematological diseases, Ageing

## Abstract

Hemophagocytic lymphohistiocytosis (HLH) is a life-threatening systemic hyper-inflammatory disorder. The mortality of HLH is higher in the elderly than in young adults. Senescence-accelerated mice (SAMP1/TA-1) exhibit characteristic accelerated aging after 30 weeks of age, and HLH-like features, including hematopoietic organ damage, are seen after lipopolysaccharide (LPS) treatment. Thus, SAMP1/TA-1 is a useful model of hematological pathophysiology in the elderly with HLH. In this study, dosing of SAMP1/TA-1 mice with LPS revealed that the suppression of myelopoiesis and B-lymphopoiesis was more severe in aged mice than in young mice. The bone marrow (BM) expression of genes encoding positive regulators of myelopoiesis (G-CSF, GM-CSF, and IL-6) and of those encoding negative regulators of B cell lymphopoiesis (TNF-α) increased in both groups, while the expression of genes encoding positive-regulators of B cell lymphopoiesis (IL-7, SDF-1, and SCF) decreased. The expression of the GM-CSF-encoding transcript was lower in aged mice than in young animals. The production of GM-CSF by cultured stromal cells after LPS treatment was also lower in aged mice than in young mice. The accumulation of the TNF-α-encoding transcript and the depletion of the IL-7-encoding transcript were prolonged in aged mice compared to young animals. LPS dosing led to a prolonged increase in the proportion of BM M1 macrophages in aged mice compared to young animals. The expression of the gene encoding p16^INK4a^ and the proportion of β-galactosidase- and phosphorylated ribosomal protein S6-positive cells were increased in cultured stromal cells from aged mice compared to those from young animals, while the proportion of Ki67-positive cells was decreased in stromal cells from aged mice. Thus, age-related deterioration of stromal cells probably causes the suppression of hematopoiesis in aged mice. This age-related latent organ dysfunction may be exacerbated in elderly people with HLH, resulting in poor prognosis.

## Introduction

Hemophagocytic lymphohistiocytosis (HLH) is a hyper-inflammatory syndrome caused by the incessant activation of lymphocytes and macrophages, resulting in organ damage of the lungs, kidneys, liver, or heart^1–3^. Patients with HLH have high levels of various proinflammatory cytokines such as interferon-γ (IFN-γ), interleukin (IL)-6, IL-8, IL-10, IL-12, IL-18, tumor necrosis factor-α (TNF-α), and macrophage inflammatory protein-1α (MIP-1α)^3^. HLH is classified largely into primary HLH (pHLH) and secondary HLH (sHLH). pHLH is caused by inherited defects in various genes (*PRF1, UNC13D, STX11*, and *STXBP2*) that regulate the granule-dependent cytotoxic pathway in NK cells and cytotoxic T cells^4–8^. pHLH also includes X-linked lymphoproliferative disease, Griselli syndrome type 2, Chediak-Higashi syndrome, and Hermansky-Pudlak syndrome^3^. In contrast, sHLH is not caused by hereditary disorder, but by severe infectious diseases, autoimmune illnesses, and malignancies^1,3,9^. Clinical manifestations of HLH include fever, splenomegaly, pancytopenia, hypertriglyceridemia and/or hypofibrinogenemia, and hemophagocytosis in bone marrow (BM), spleen, or lymph nodes, accompanied by low or absent NK cells, hyperferritinemia, and high soluble levels of IL-2 receptor^1^. sHLH usually occurs in young adolescents but has been reported in the elderly, albeit only rarely^10,11^. Although it has been reported that the prognosis of patients with sHLH is poorer in the elderly than in young adults, little is known about the pathogenesis and the pathophysiology that lead to worse prognoses among elderly patients with sHLH^10,12–14^.

Previously, we demonstrated that SAMP1/TA-1 mice exhibit a senescent hematological phenotype including senescence-like stromal cell impairments after 30 weeks of age^15–19^. We recently found that SAMP1/TA-1 mice dosed repeatedly with lipopolysaccharide (LPS) exhibit HLH-like features such as severe pancytopenia, hepatomegaly, splenomegaly, hypofibrinogenemia, hyperferritinemia, and hemophagocytosis in peripheral blood, BM, and spleen; thus, they serve as a useful model of sHLH^20^. In the BM of SAMP1/TA-1 mice repeatedly treated with LPS, B lymphoid progenitor cells are persistently and profoundly decreased. The mRNA levels of positive regulators of B lymphopoiesis, such as IL-7, stromal-cell derived factor-1 (SDF-1), and stem cell factor (SCF) are also profoundly decreased in both the BM and cultured stromal cells^21^. Furthermore, senescence markers, such as the expression of the gene encoding p16^INK4a^ and the proportion of β-galactosidase-positive cells, are increased in cultured stromal cells^21^. There are similarities between hematopoietic alterations during inflammation and those that occur with aging. The cause of pancytopenia in HLH has previously been explained by the unregulated production of circulating proinflammatory cytokines including TNF-α and IFN-γ, possibly produced by activated T lymphocytes, monocytes/macrophages, and endothelial cells^3^. However, our recent study revealed that prolonged hyper-inflammation in HLH severely deteriorates the stromal cells that comprise the microenvironment that supports hematopoiesis in BM, resulting in disrupted dynamics of that hematopoiesis^21^.

Thus, the dynamics of hematopoiesis in the BM are disrupted by an impairment of the hematopoietic microenvironment in LPS-dosed SAMP1/TA-1 mice, indicating that the hematopoietic tissue is among the organs that suffer life-threatening damage in HLH^21^. Therefore, we chose the hematopoietic organ to investigate differences in the pathophysiology of sHLH between young and elderly adults.

Hematological parameters that reflect hematopoietic dynamics (such as the number of peripheral blood cells and hematopoietic progenitor cells in the BM) and the levels (in the hematopoietic microenvironment) of mRNAs encoding hematopoiesis regulatory cytokines should be good indicators for evaluating the pathophysiology that exacerbates organ damage in elderly patients with HLH^20,21^. In the present study, we compared hematological parameters between young (8- to 12-week-old) and aged (30- to 36-week-old) SAMP1/TA-1 mice dosed with LPS.

## Results

### Changes in the numbers of peripheral blood cells in young and aged SAMP1/TA-1 mice after LPS treatment

**Figure 1:**
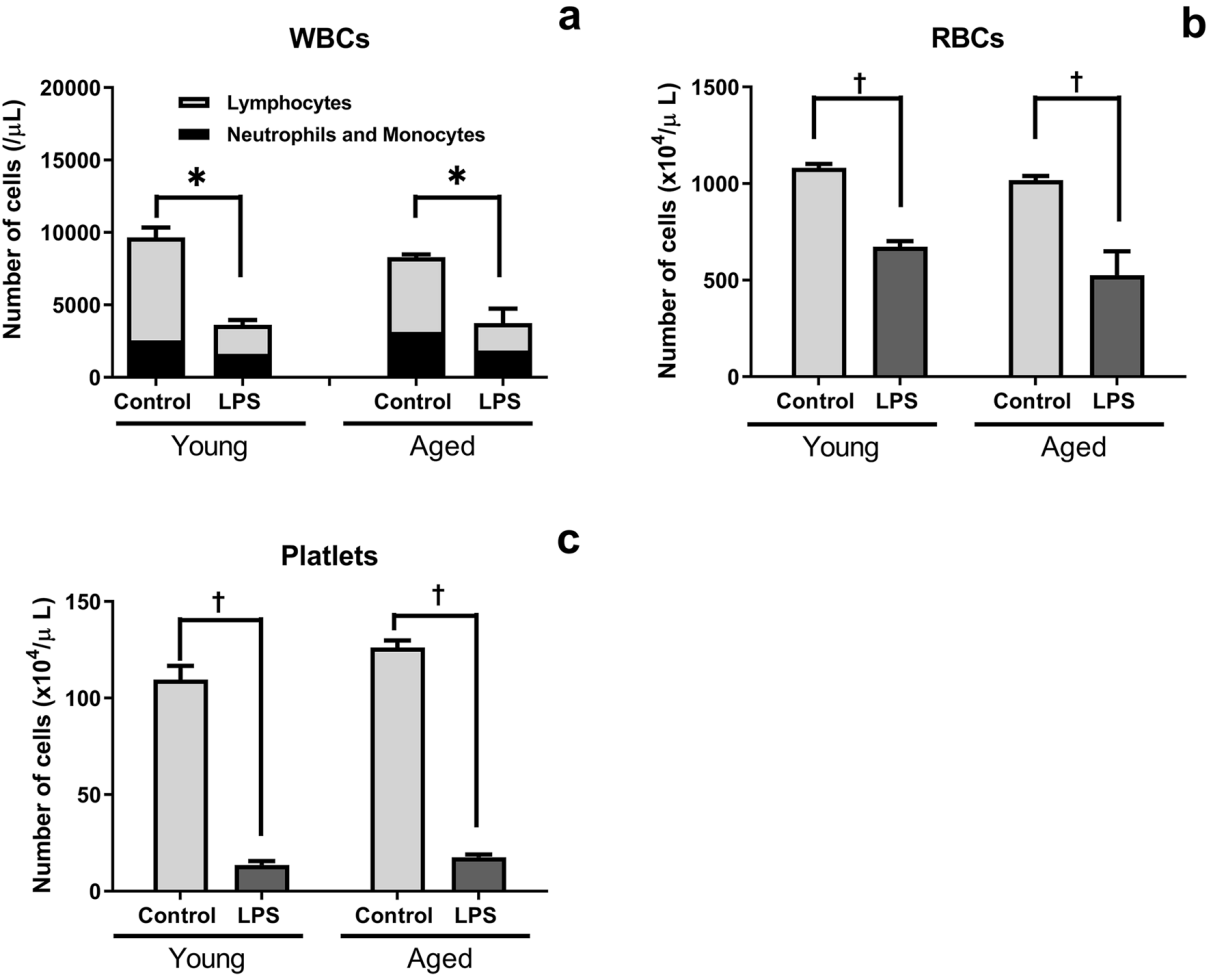
Changes in the numbers of peripheral blood cells after LPS treatment. Changes in the numbers of WBCs, RBCs, and platelets in young and aged SAMP1/TA-1 mice after LPS treatment are shown. Changes in the number of WBCs (**a**), RBCs (**b**), and platelets (**c**) in young and aged SAMP1/TA-1 mice after intravenous injection of 25 μg LPS are shown. Samples of peripheral blood cells were obtained from three mice per group at nine days after treatment with saline or LPS. Each bar represents the mean ± SD. *P < 0.05, ^†^P < 0.005 vs. saline-treated control.

The numbers of peripheral white blood cells (WBCs), red blood cells (RBCs), and platelets were evaluated in the blood of young and aged SAMP1/TA-1 mice nine days after dosing with saline (control) or LPS (Fig. 1). The number of WBCs in non-treated young and aged mice was 9695 ± 650/µl and 8299 ± 173/µl, respectively. The number of WBCs in young and aged mice after LPS treatment was significantly decreased to 37.6% and 43.4% those of control mice, respectively (Fig. 1a). The number of RBCs in non-treated young and aged mice was 1081 ± 20 × 10^4^/µl and 1018 ± 20 × 10^4^/µl, respectively. The number of RBCs in young and aged mice after LPS treatment was significantly decreased to 62.2% and 51.6% those of control mice, respectively (Fig. 1b). The number of platelets in non-treated young and aged mice was 110 ± 7 × 10^4^/µl and 126 ± 4 × 10^4^/µl, respectively. The number of platelets in young and aged mice after LPS treatment was significantly decreased to 12.4% and 13.9% those of control mice, respectively (Fig. 1c). There were no significant differences in the numbers of WBCs, RBCs, or platelets between LPS-treated young mice and LPS-treated aged mice.

### Changes in the numbers of hematopoietic progenitor cells in the BM of young and aged SAMP1/TA-1 mice after LPS treatment

**Figure 2:**
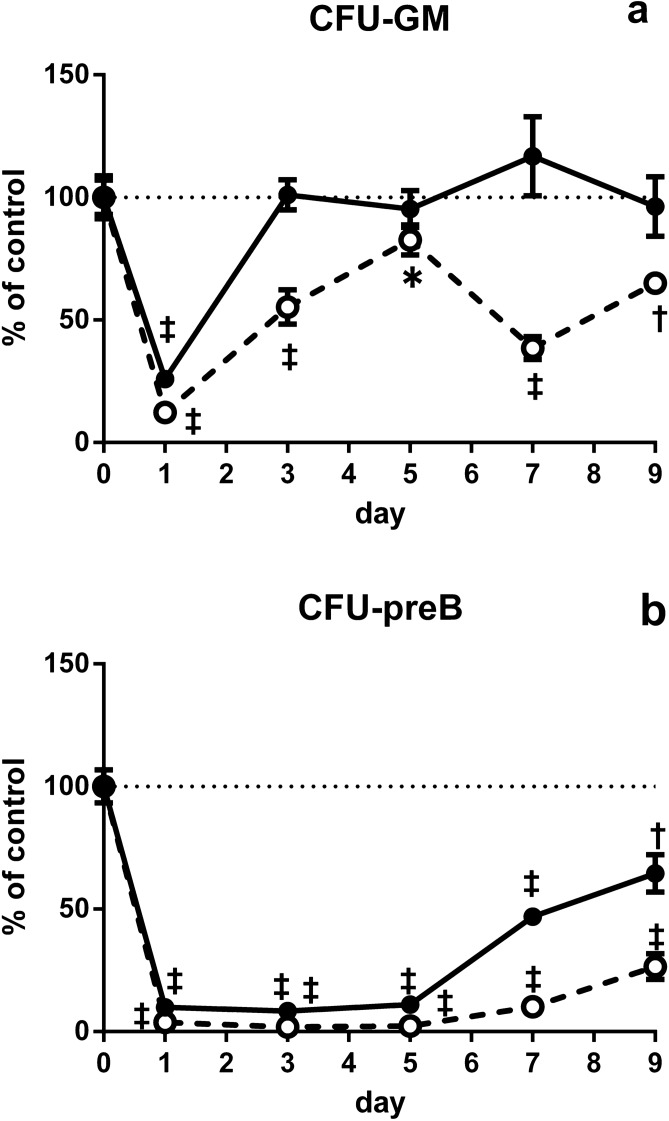
Changes in the numbers of hematopoietic progenitor cells in BM after LPS treatment. Changes in the numbers of hematopoietic progenitor cells in the BM of young and aged SAMP1/TA-1 mice after LPS treatment are shown. Changes in the numbers of CFU-GM (**a**) and CFU-preB (**b**) in young (closed circles) and aged (open circles) SAMP1/TA-1 mice after intravenous injection of 25 μg LPS are shown. Samples of femoral BM were obtained from three mice per group at each time point (1, 3, 5, 7, and 9 days) after treatment with LPS. The results are expressed as the percentage of control. Each bar represents the mean ± S.D. *P < 0.05, ^†^P < 0.005, ^‡^P < 0.001 vs. saline-treated control.

The numbers of CFU-GM and CFU-preB cells in the BM were evaluated in young and aged SAMP1/TA-1 mice after LPS treatment (Fig. 2). The number of CFU-GM in non-treated young- and aged-SAMP1/TA-1 mice was 41,920 ± 2963 and 63,419 ± 5411, respectively. The number of CFU-GM in young SAMP1/TA-1 mice after LPS treatment decreased rapidly to 26% of the pretreatment level during the first 24 h, increased to 101% of the pretreatment level by day 3, and remained unchanged thereafter. The number of CFU-GM in aged SAMP1/TA-1 mice after LPS treatment decreased rapidly to 12% of the pretreatment level during the first 24 h, increased to 82% of the pretreatment level by day 5, and remained lower than the pretreatment levels thereafter. The numbers of CFU-GM at days 3, 7, and 9 after LPS treatment were significantly lower in aged mice than in young mice (P < 0.05). The number of CFU-preB cells in non-treated young and aged SAMP1/TA-1 mice was 15,171 ± 1037 and 11,222 ± 602, respectively. The number of CFU-preB cells in young SAMP1/TA-1 mice after LPS treatment decreased rapidly to 8% of the pretreatment level by day 3 and increased to 65% of the pretreatment level by day 9. The number of CFU-preB cells in aged SAMP1/TA-1 mice after LPS treatment decreased rapidly to 2% of the pretreatment level by day 3, subsequently rising to only 27% of the pretreatment level by day 9. The numbers of CFU-preB at days 7 and 9 after LPS treatment were significantly lower in aged mice than in young mice (P < 0.05).

### Changes in the proportion and the number of hematopoietic progenitor cells in S phase in the BM of young and aged SAMP1/TA-1 mice after LPS treatment

**Figure 3:**
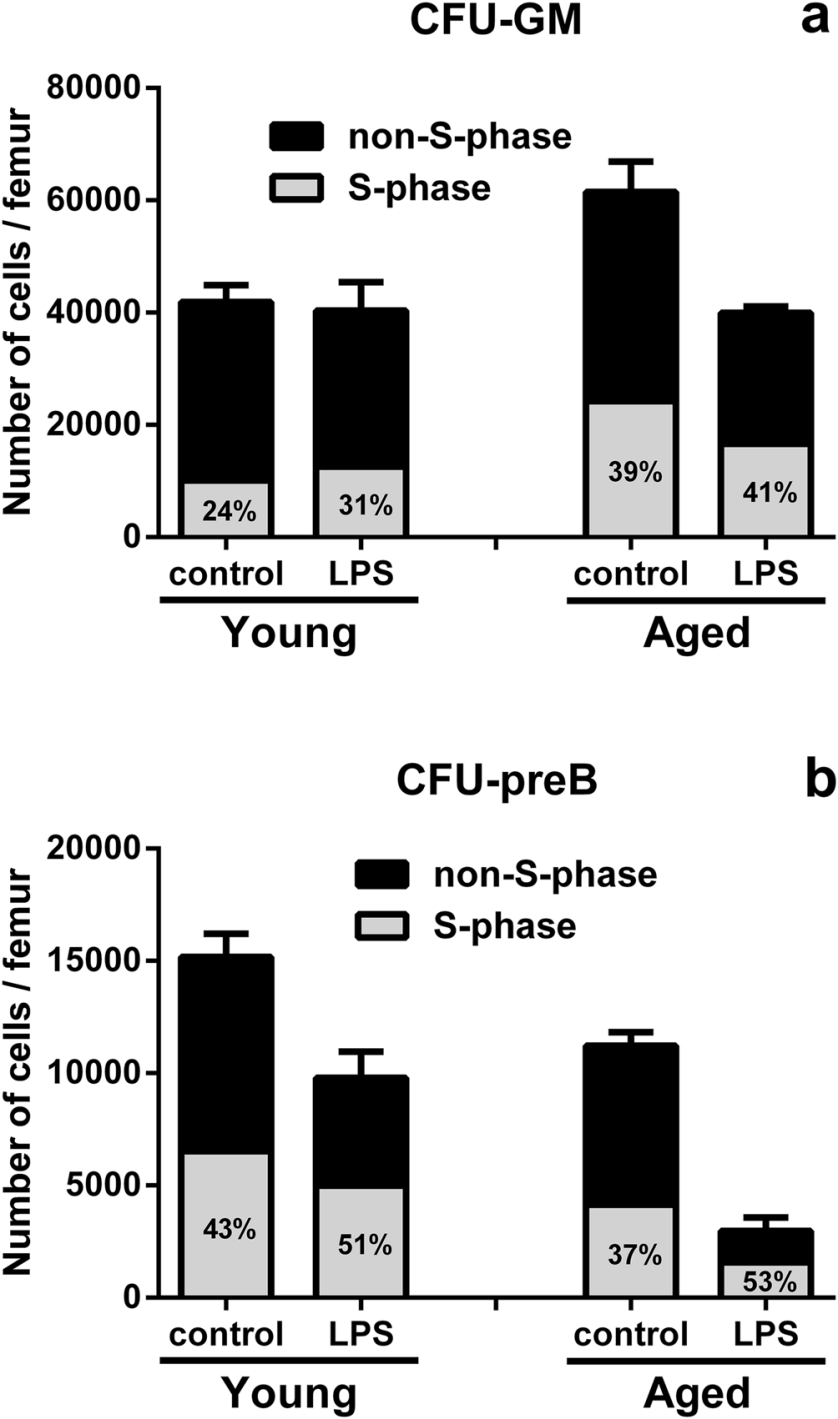
Changes in the proportion and the number of hematopoietic progenitor cells in S phase in the BM after LPS treatment. Changes in the proportion and the number of hematopoietic progenitor cells in S phase in the BM of young and aged SAMP1/TA-1 mice after LPS treatment are shown. The numbers (absolute and proportional) of S-phase CFU-GM (**a**) and CFU-preB (**b**) cells in young and aged SAMP1/TA-1 mice nine days after treatment with saline or 25 µg LPS are shown. The proportions of S phase CFU-GM and CFU-preB cells in young and aged mice are indicated as percentages in the bars. Femoral BM samples were obtained from three mice per group at nine days after treatment with saline or 25 µg LPS. Each bar represents the mean ± S.D.

Changes in the numbers of CFU-GM and CFU-preB cells in S phase in the BM were evaluated in young and aged SAMP1/TA-1 mice nine days after LPS treatment, as assessed using cell suicide induced by exposure to a low concentration of hydroxyurea (HU) (Fig. 3). The proportions of CFU-GM cells in S phase of saline-treated (control) and LPS-treated young mice were 24 ± 4% and 31 ± 2%, respectively (Fig. 3a). The proportions of CFU-GM cells in S phase of saline-treated (control) and LPS-treated aged mice were 39 ± 4% and 41 ± 2%, respectively (Fig. 3a). The number of CFU-GM cells in S phase of non-treated (control) and LPS-treated young mice was 10,061 ± 1676 and 12,513 ± 807, respectively (Fig. 3a). The number of CFU-GM cells in S phase of non-treated (control) and LPS-treated aged mice was 24,733 ± 2536 and 16,903 ± 824, respectively (Fig. 3a).

The proportions of CFU-preB cells in S phase of non-treated (control) and LPS-treated young mice were 43 ± 1% and 51 ± 4%, respectively (Fig. 3b). The proportions of CFU-preB cells in S phase of non-treated (control) and LPS-treated aged mice were 37 ± 7% and 53 ± 6%, respectively (Fig. 3b). The number of CFU-preB cells in S phase of non-treated (control) and LPS-treated young mice was 6523 ± 151 and 4997 ± 391, respectively (Fig. 3b). The number of CFU-preB cells in S phase of non-treated (control) and LPS-treated aged mice on day 9 after LPS treatment was 4152 ± 785 and 1576 ± 208, respectively (Fig. 3b).

### Changes in levels of transcripts encoding regulatory cytokines in the BM of young and aged SAMP1/TA-1 mice after LPS treatment

**Figure 4:**
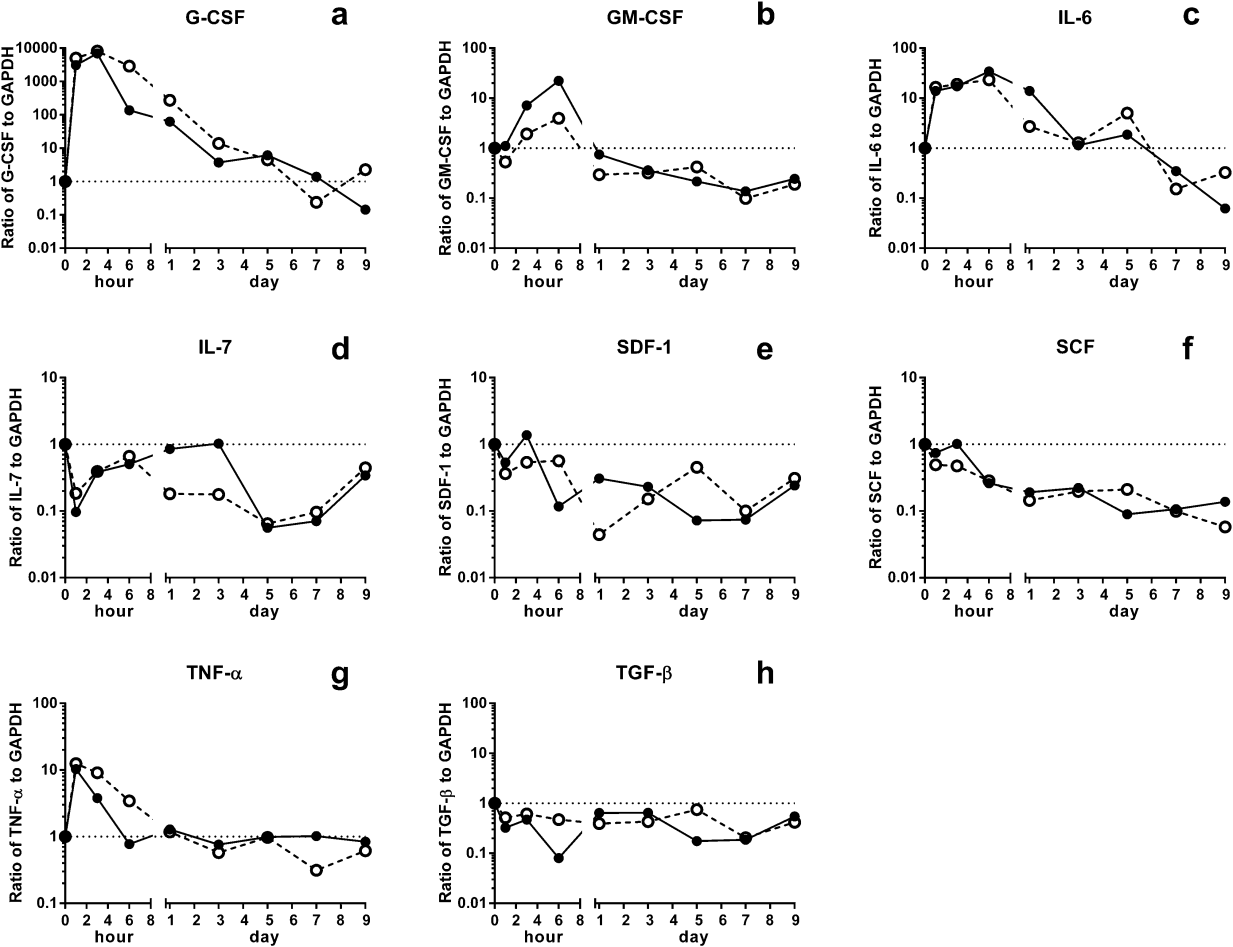
Changes in levels of transcripts encoding regulatory cytokines in the BM after LPS treatment. Changes in the relative levels of transcripts encoding G-CSF, GM-CSF, IL-6, IL-7, SDF-1, SCF, TNF-α, and TGF-β in the BM of young and aged SAMP1/TA-1 mice after LPS treatment are shown. The levels of transcripts encoding positive regulators of myelopoiesis G-CSF (**a**), GM-CSF (**b**), and IL-6 (**c**); encoding positive regulators of B lymphopoiesis IL-7 (**d**), SDF-1 (**e**), and SCF (**f**); and encoding negative regulators of B lymphopoiesis TNF-α (**g**) and TGF-β (**h**) were evaluated in young (closed circles) and aged (open circles) SAMP1/TA-1 mice at 1, 3, and 6 h and at 1, 3, 5, 7, and 9 days after treatment with 25 μg LPS are shown. Values shown for young and aged SAMP1/TA-1 mice after LPS treatment are normalized to the levels in non-treated control young and aged SAMP1/TA-1 mice, respectively. Each bar represents the mean ± S.D. The values in non-treated young and aged SAMP1/TA-1 mice were arbitrarily set to a value of 1.

The hematopoietic microenvironment is composed of BM stromal cells that regulate hematopoiesis by producing positive and negative regulators. G-CSF, GM-CSF, and IL-6 are positive regulators of myelopoiesis. IL-7, SDF-1, and SCF are positive regulators of B lymphopoiesis, while TNF-α and TGF-β are negative regulators of B lymphopoiesis. All of these cytokines are known to be produced by stromal cells in BM^21–23^. To examine the LPS-induced changes in the regulation of myelopoiesis and B lymphopoiesis by BM stromal cells, we evaluated the levels of transcripts encoding regulators of myelopoiesis and B lymphopoiesis. For positive regulators of myelopoiesis, the levels of G-CSF, GM-CSF, and IL-6 were highly increased in BM cells from both young and aged SAMP1/TA-1 mice at 1, 3, and 6 h after LPS treatment, followed by rapid decreases (Fig. 4a–c). The magnitude of increase of the levels of mRNA encoding GM-CSF at 3 and 6 h after LPS treatment was significantly smaller in aged mice than in young mice (P < 0.05) (Fig. 4b). For positive regulators of B lymphopoiesis, BM cells from both young and aged SAMP1/TA-1 mice exhibited decreases in the levels of transcripts encoding IL-7, SDF-1, and SCF (Fig. 4d–f). The levels of mRNA encoding IL-7 in the BM of LPS-treated aged mice from day 1 through day 3 were significantly lower than those in young mice (P < 0.05) (Fig. 4d). For negative regulators of B lymphopoiesis, BM cells from both young and aged SAMP1/TA-1 mice exhibited a marked increase in the levels of transcripts encoding TNF-α and a decrease in the levels of transcripts encoding TGF-β (Fig. 4g,h). The levels of mRNA encoding TNF-α in the BM of LPS-treated aged mice from 3 through 6 h after dosing were significantly higher than those in young mice (P < 0.05) (Fig. 4g).

### Levels of GM-CSF and TNF-α produced by cultured BM stromal cells from young and aged SAMP1/TA-1 mice

**Figure 5:**
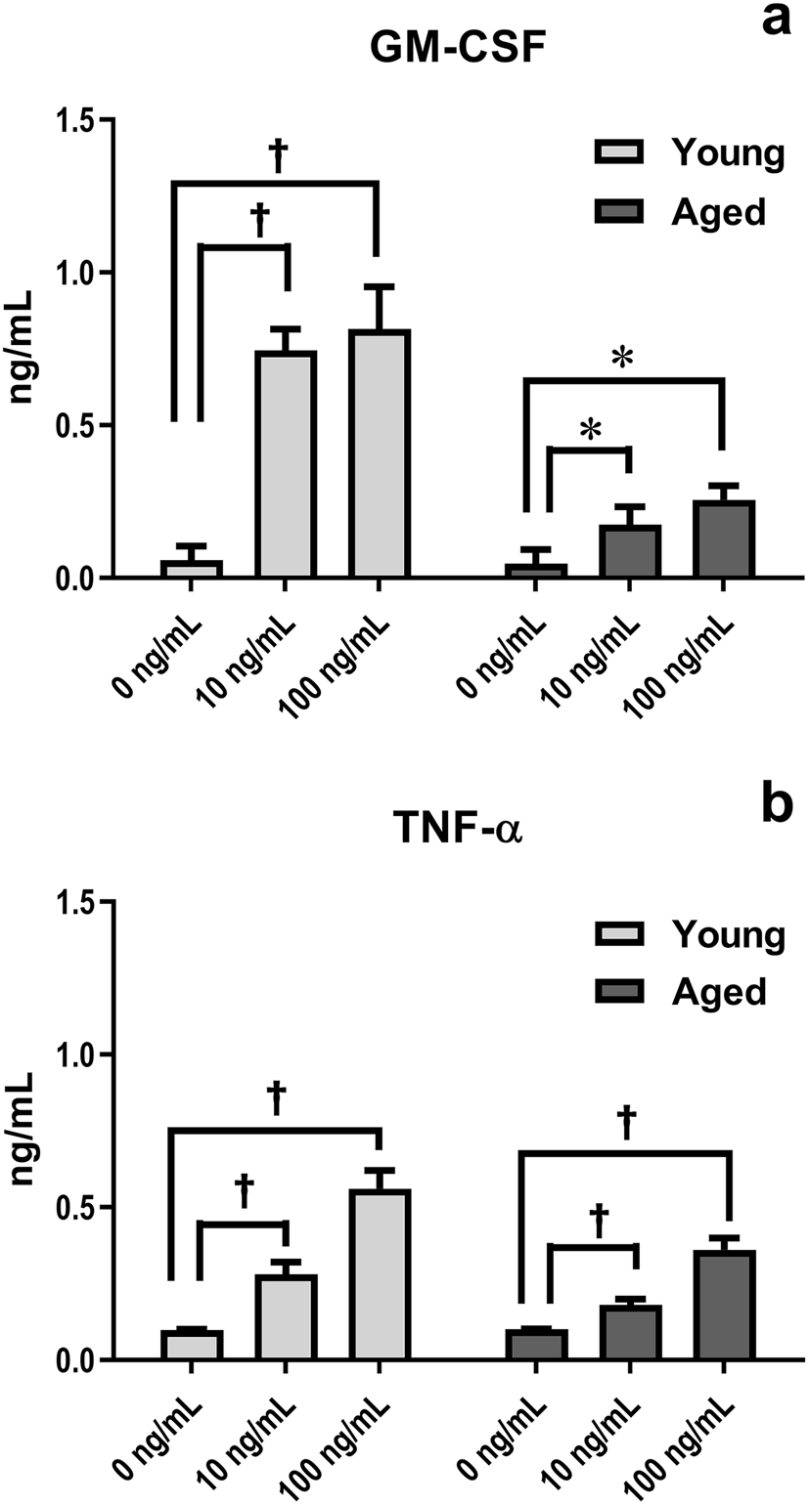
Cytokine production by cultured stromal cells after LPS treatment. The productions of GM-CSF (**a**) and TNF-α (**b**) by cultured stromal cells obtained from young and aged mice after LPS treatment are shown. The concentrations of GM-CSF and TNF-α in the culture medium were determined using GM-CSF- and TNF-α-specific ELISA. Each bar represents the mean ± SD obtained from three culture wells. *P < 0.05, ^†^P < 0.005.

To study differences in stromal cell function between young and aged SAMP1/TA-1 mice, the production of GM-CSF and TNF-α by cultured stromal cells from young and aged mice was examined (Fig. 5). In both young and aged mice, LPS treatment induced the production of GM-CSF and TNF-α in an LPS concentration-dependent manner. The production of TNF-α by stromal cells established from young mice was higher than that from aged mice; however, a significant difference was not observed. In contrast, a slight but significant difference (P < 0.05) was observed in the production of GM-CSF between young and aged mice after LPS treatment.

### Changes in the polarization of M1/M2 macrophages in the BM of young and aged SAMP1/TA-1 mice after LPS treatment

**Figure 6:**
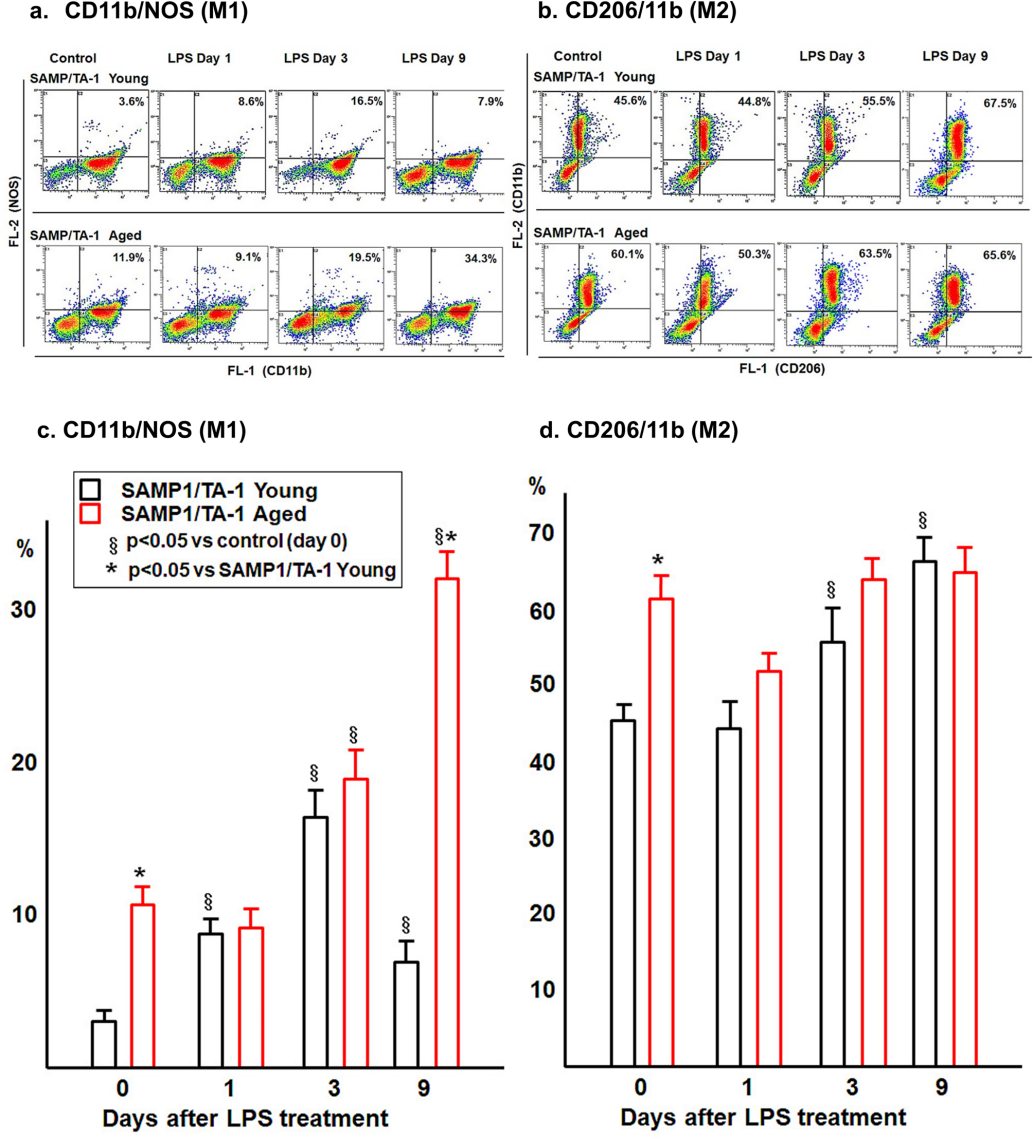
The proportions of M1 and M2 macrophages in BM of young and aged mice. The proportions of M1 and M2 macrophages in the BM of young and aged SAMP1/TA-1 mice after LPS treatment are shown. Typical dot plot histograms of two-color flow cytometry determined by CD11b (FL-1) and NOS (FL-2) for detecting M1 macrophages (**a**) and by CD206 (FL-1) and CD11b (FL-2) for detecting M2 macrophages (**b**) are shown. Changes in the proportions of M1 and M2 cells after LPS treatment are shown in (**c**) and (**d**). The samples of macrophages of the BM were obtained from non-treated control mice (day 0) and from treated mice at 1, 3, and 9 days after dosing with 25 µg of LPS. Each bar represents the mean ± SD. ^§^P < 0.05 vs. control (day 0) and *P < 0.05 vs. SAMP1/TA-1 young mice.

The polarization of M1/M2 macrophages was evaluated in the BM of young and aged SAMP1/TA-1 mice after LPS treatment. Figure 6 shows the changes in the proportions of M1 and M2 macrophages in young and aged SAMP1/TA-1 mice after LPS treatment. In the control mice, the proportions of M1 macrophages (CD11b-positive/NOS-positive cells) (Fig. 6a,c) and M2 macrophages (CD11b-positive/CD206-positive cells) (Fig. 6b,d) were higher in aged mice than in young mice. The proportion of M2 macrophages was higher than that of M1 macrophages in both young and aged SAMP1/TA-1 mice before LPS treatment (Fig. 6c,d). When treated with LPS, the proportion of M1 macrophages in young mice was increased by day 3, followed by a decrease at day 9 (Fig. 6c). However, the proportion of M1 macrophages in aged mice continued to increase through day 9 (Fig. 6c). After treatment with LPS, the proportion of M2 macrophages in young mice gradually and continuously increased through day 9 (from 45.6 ± 5.2% to 67.5 ± 4.4%; P < 0.05) (Fig. 6d). However, the proportion of M2 macrophages in aged mice showed temporality and insignificantly fell by day 1 after LPS treatment (from 60.1 ± 5.2% to 50.3 ± 4.0%), followed by a subsequent increase to pretreatment levels at day 3 (63.5 ± 5.0%) (Fig. 6d).

### Age-related qualitative changes of BM stromal cells in SAMP1/TA-1 mice

**Figure 7:**
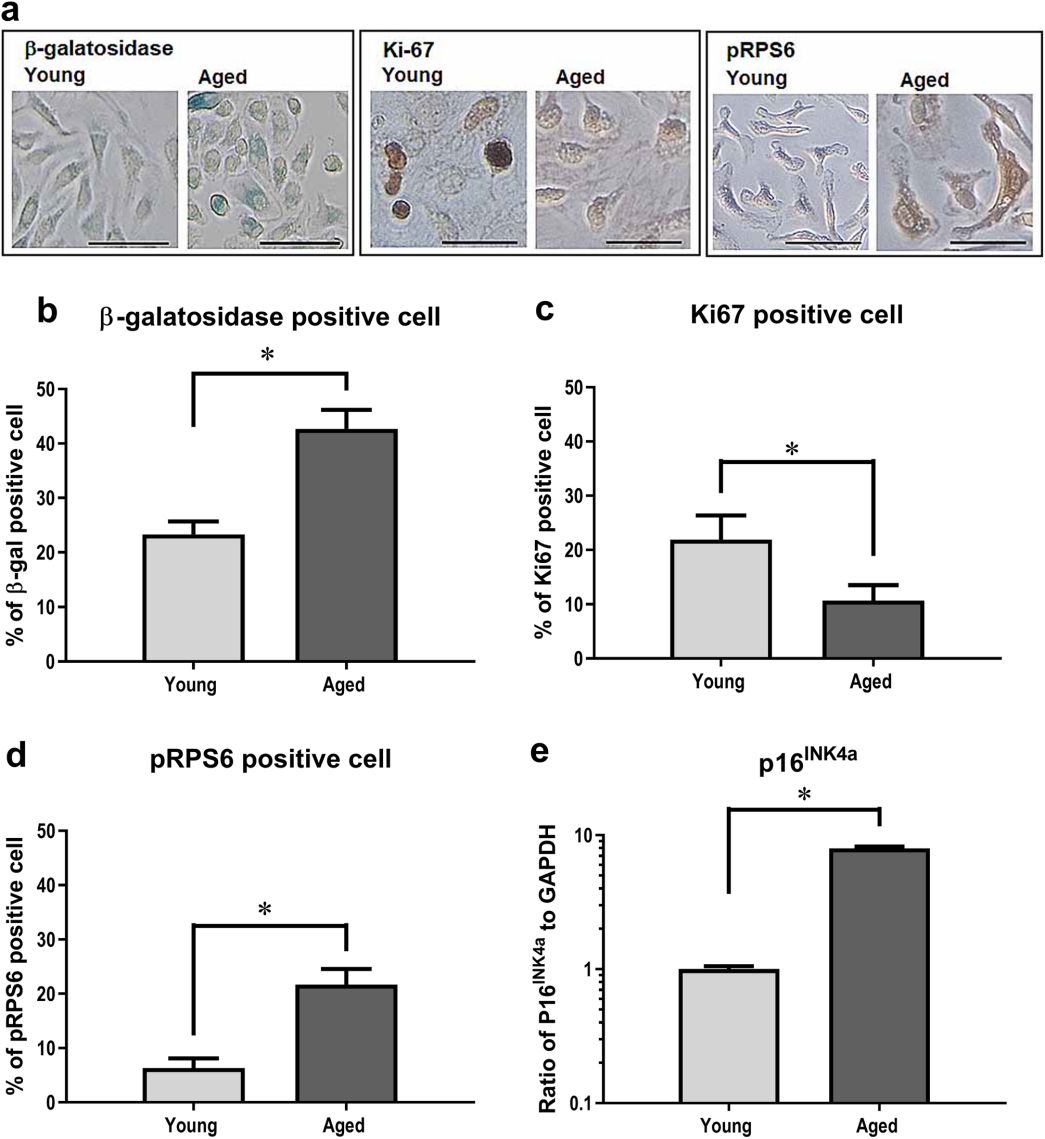
Age-related qualitative changes in BM stromal cells. (**a**) Images of cultured stromal cells obtained from young and aged SAMP1/TA-1 mice. β-galactosidase-positive cells are indicated by blue staining. Ki67-positive cells were identified by brown staining in nuclei, and pRPS6-positive cells were identified by brown staining in the cytoplasm. Scale bar, 50 µm. The bar graph shows proportions of β-galactosidase-positive cells from (**b**), Ki-67-positive cells from (**c**), and pRPS6-positive cells from (**d**). (**e**) Levels of p16^INK4a^-encoding transcripts in cultured stromal cells obtained from young and aged SAMP1/TA-1 mice. The values in aged mice are normalized to the level in young mice, which was arbitrarily set to a value of 1. Each bar represents the mean ± SD obtained from three culture wells. *P < 0.05.

To characterize age-related qualitative changes in BM stromal cells, we evaluated the proportions of β-galactosidase-, Ki67-, and phosphorylated ribosomal protein S6 (pRPS6) -positive cells and the levels of transcripts encoding p16^INK4a^ in cultured stromal cells in young and aged SAMP1/TA-1 mice (Fig. 7). Figure 7a shows the images of β-galactosidase-, Ki67-, and pRPS6-positive cultured stromal cells obtained from young and aged SAMP1/TA-1 mice. The proportions of β-galactosidase-positive cells among cultured stromal cells obtained from non-treated control young and aged SAMP1/TA-1 mice were 23.3% and 42.7%, respectively (Fig. 7b). It has been reported that β-galactosidase activity is increased not only in senescent cells but also in stressed cells. A definitive senescent phenotype can be identified by evaluating the expressions of Ki67, pRPS6, and β-galactosidase^24^. Thus, we also evaluated the proportions of Ki67- and pRPS6-positive cells in cultured stromal cells in young and aged SAMP1/TA-1 mice. The proportions of Ki67-positive cells in cultured stromal cells obtained from saline-treated control young and aged SAMP1/TA-1 mice were 22% and 11%, respectively (Fig. 7c). The proportions of pRPS6-positive cells in cultured stromal cells from saline-treated control young and aged SAMP1/TA-1 mice were 6% and 22%, respectively (Fig. 7d). The level of the mRNA encoding p16^INK4a^ in cultured stromal cells from aged SAMP1/TA-1 mice was 796% of that from young SAMP1/TA-1 mice (Fig. 7e).

## Discussion

Overproduction of proinflammatory cytokines is seen in sHLH, but the mechanisms underlying this hypercytokinemia are not clear^3^. An sHLH-like syndrome in a mouse model can be induced using a ligand for toll-like receptor (TLR), such as cytosine guanine dinucleotides and LPS^20,21,25–27^. Thus, the overproduction of proinflammatory mediators may be because of sustained TLR activation by infection or an autoimmune trigger^28^. TLR4 for the ligand LPS is expressed not only on hematopoietic cells, but also on non-hematopoietic cells such as stromal cells. However, it has been reported that the primary, indispensable in vivo sensing site for LPS-induced emergency myelopoiesis is a TLR4-expressing nonhematopoietic cellular compartment, such as stromal cells in the BM^29^. The present results support the idea that BM stromal cells play a key role in the hematological changes in HLH.

With aging, hematopoietic defects become particularly evident in the lymphoid linages, and can be attributed to the skewing of the hematopoietic stem cell (HSC) repertoire toward the myeloid lineage, which suggests dynamic hematopoiesis during steady-state changes with aging^17,30,31^. Hematopoiesis in the BM is strictly regulated by stromal cells composed of the hematopoietic microenvironment via diffusible factors and direct cellular interactions via adhesion molecules^32–34^. Current studies are revealing that the hematopoietic microenvironment may contribute to HSC aging^17,35^. The age-related change in hematopoiesis is latent and not harmful in the ordinary lives of the elderly. However, HLH is a life-threatening systemic hyper-inflammatory disorder trigged by a prolonged and tremendous cytokine storm^1,2^. Thus, we postulate that latent deterioration of the hematopoietic organ may be revealed and exacerbated in elderly patients with HLH.

Inflammation redirects central hematopoiesis by altering the hematopoietic microenvironment to favor myelopoiesis over lymphopoiesis, thereby inducing the mobilization of myeloid cells and B lymphoid cells followed by an acceleration of myelopoiesis to replenish the mature, consumed neutrophils and a concomitant inhibition of B lymphopoiesis^36^. When SAMP1/TA-1 mice were dosed with LPS, the numbers of CFU-GM and CFU-preB cells in both young and aged animals rapidly decreased. The recovery from the nadir in the number of CFU-GM cells was prompt. In contrast, the recovery in the number of CFU-preB cells from the nadir was delayed (Fig. 2). These results are compatible with those of previous studies^21–23^. Interestingly, the recoveries from the nadir in the number of not only CFU-preB cells but also CFU-GM cells in aged mice were limited compared with those in young mice. In myelopoiesis, the proportion of cycling CFU-GM cells in saline-treated (control) aged mice was higher than that in control young mice; this result suggested that myelopoiesis under steady-state conditions is accelerated with aging, as previously reported^37,38^. Although the total number of CFU-GM cells in aged mice on day 9 after LPS treatment was lower than that in control animals, the proportion of cycling CFU-GM cells in aged mice on day 9 after LPS treatment was about the same as that in control animals (Fig. 3a); this observation indicated the kinetics of myelopoiesis in aged mice on day 9 after LPS treatment was not hyperdynamic but normodynamic. Additionally, the magnitude of accumulation of transcripts encoding positive regulatory cytokines (such as GM-CSF) during the first 3 h after LPS treatment was smaller in aged mice than in young mice (Fig. 4b). Taken together, these data suggest that the LPS-induced positive regulation of myelopoiesis by stromal cells is impaired in aged mice. In B lymphopoiesis, the proportion of cycling CFU-preB cells in young and aged mice on day 9 after LPS treatment was increased compared with that in control mice (Fig. 3b). These results suggested that B lymphopoiesis of young and aged mice is accelerated on day 9 after LPS treatment. The total number of cycling CFU-preB cells on day 9 after LPS treatment was more attenuated in aged mice than in young mice (Fig. 3b). Additionally, the accumulation of transcripts encoding negative regulators (such as TNF-α) and the depletion of transcripts encoding positive regulators (such as IL-7) in aged mice were prolonged compared with those in young mice (Fig. 4d,g). Taken together, these data suggested that the recovery in the number of CFU-preB cells after LPS treatment is delayed in aged mice compared with that in young mice; presumably, an imbalance in the positive and negative regulation of B lymphopoiesis by stromal cells during the recovery period may occur in aged mice. In addition, the production and release of GM-CSF and TNF-α from LPS-stimulated stromal cells in vitro (48 h after LPS stimulation) were studied by ELISA (Fig. 5). Only GM-CSF in stromal cells from young mice was produced at a significantly higher level than from aged mice after LPS treatment. However, the difference in the GM-CSF level between young and aged mice was small (0.81 ng/mL in young and 0.26 ng/mL in aged after 100 ng/mL LPS stimulation), which may also suggest some lack of stromal cell function in aged mice.

Macrophages commonly exist in two distinct subsets, M1 and M2. M1 and M2 macrophages have opposing functions: M1 macrophages are proinflammatory, and M2 macrophages are anti-inflammatory, and the M1/M2 macrophage balance governs the inflammation process^39^. Interestingly, the proportion of both M1 and M2 macrophages of aged mice in the control was higher than that of macrophages in young mice. These results suggest that inflammation happens chronically in aged mice even if the mice were not treated with LPS. In addition, LPS treatment resulted in a prolonged increase in the proportion of M1 macrophages in aged mice (compared to that in young mice) as measured on day 9 after LPS treatment (Fig. 6). These data indicated further and stronger inflammation that is prolonged in the BM of aged mice compared to that in younger animals. The importance of macrophage polarization has been previously reported; Wang et al. showed that an imbalance of M1 and M2 macrophage polarization is associated with inflammation and various disorders^27^. Thus, LPS treatment led to severely unbalanced M1 and M2 macrophage polarization, causing serious and persistent inflammation in aged mice.

The levels of the transcript encoding p16^INK4a^ and the proportions of β-galactosidase- and pRPS6-positive cells were higher in cultured stromal cells obtained from aged mice than in those obtained from young mice. In contrast, the proportion of Ki67-positive cells was lower in cultured stromal cells obtained from aged mice than in those obtained from young mice (Fig. 7). Both p16^INK4a^ and β-galactosidase are well-known markers of senescence^40^. Furthermore, Alessio et al. recently identified three phenotypes: Ki67(+) pRPS6(+) β-galactosidase(+) cells, which are referred to as stress cells; Ki67(+) pRPS6(−) β-galactosidase(+) cells and Ki67(−) pRPS6(−) β-galactosidase(+) cells, which are referred to as pre-senescent; and Ki67(−) pRPS6(+) β-galactosidase(+) cells, which are referred to as senescent^24^. Taken together, our results indicated that cellular senescence occurred in the BM stromal cells of SAMP1/TA-1 mice with aging.

The functions of organs other than hematopoietic tissue also gradually deteriorate with aging^41^. Thus, the latent dysfunction of organs may progress rapidly, which may explain the poor prognosis observed for elderly patients with HLH.

## Methods

### Mice

SAMP1/TA-1 mice were bred and maintained in an experimental facility at the Nihon University School of Medicine^15,16^. Eight- to twelve-week-old (young) and thirty- to 36-week-old (aged) SAMP1/TA-1 male mice were used. For each data point of the protocol, three mice were examined (n = 3) to avoid emphasizing exceptional results. All protocols involving laboratory mice were reviewed and approved by the Nihon University Animal Care and Use Committee (Experimental Codes AP19MED019-2 and AP19MED050-1). The approved experimental protocol was performed humanely in strict accordance with ARRIVE criteria and the Nihon University “Rules Concerning Animal Care and Use”.

### LPS treatment

*Escherichia coli* LPS055:B5 (Sigma Chemical Co., St. Louis, MO, USA) was diluted in pyrogen-free saline to a final concentration of 125 µg/mL, and mice were injected intravenously at 25 µg/animal^20,21^. A control group of young and aged SAMP1/TA-1 mice was dosed with the same volume of pyrogen-free saline.

### Preparation of peripheral blood cells and femoral BM cells

Peripheral blood was collected from the retro-orbital plexus of mice under isoflurane anesthesia. Peripheral blood was smeared on glass slides, stained with Wright-Giemsa reagent, and then 100 cells were counted differentially according to the type of WBC^20,21^. BM cell suspensions were prepared by repeatedly flushing the cells from femora using either Iscove-modified Dulbecco’s medium (IMDM: Invitrogen Corp., Carlsbad, CA, USA) or α-minimal essential medium (α-MEM: Thermo Fisher Scientific, Waltham, MA, USA); the cells then were dispersed by repeated passage through a 23-gauge hypodermic needle. BM cells were recovered from the femora of three mice per experimental group at each time point; the resulting individual cell suspensions then were counted. The number of cells was determined using a Sysmex PocH-100 *i*V Diff hematology analyzer (Sysmex Co., Kobe, Japan).

### Progenitor cell colony assay

The method used for the assay of hematopoietic progenitors has been described in detail elsewhere^20,21^. In brief, myeloid progenitor cells (CFU-GM) were assayed using MethoCult M3231 (Stem Cell Technologies, Inc., Vancouver, BC, Canada) supplemented with 10 ng/mL recombinant murine granulocyte–macrophage colony-stimulating factor (rmGM-CSF) (R&D Systems, Minneapolis, MN, USA). B-lymphoid progenitor cells (CFU-preB) were assayed using MethoCult M3630 (Stem Cell Technologies, Inc.). Cells were cultured in a humidified incubator at 37 °C and 5% CO_2_. CFU-GM and CFU-preB cells were counted seven days after plating the cells.

### Cell suicide experiments by hydroxyurea (HU) to count hematopoietic progenitor cells in S phase

Equal volumes of a cell suspension were placed into two test tubes. HU (Sigma-Aldrich, Inc., St. Louis, MO, USA) was dissolved in IMDM and added to one tube to a final concentration of 6 mM^19,22^. IMDM alone was added to the control. Both tubes were incubated for 1 h at 37 °C. After washing three times with IMDM, CFU-GM and CFU-preB cell assays were performed. CFU-GM and CFU-preB cells in S phase are selectively killed by HU. Thus, the proportions of cells in S phase among CFU-GM and CFU-preB cells were calculated using the following formula: (untreated colony number − HU-treated colony number)/untreated colony number.

### Flow cytometry analysis for M1 and M2 macrophage polarization in the BM of young and aged SAMP1/TA-1 mice

Harvested bone marrow cells were washed with PBS and passed through a 35-μm filter (Cell Strainer; Falcon 352235, Becton Dickinson Labware, Franklin Lake NJ, USA) to remove the bone debris and aggregated cells. Cells (2 × 10^6^) were suspended in 0.5 mL PBS and incubated with FITC-conjugated rat anti-mouse CD11b monoclonal antibody (BD Pharmingen; Clone M1/70, Material number 557396) or PE-conjugated rat anti-mouse CD11b monoclonal antibody (BD Pharmingen; Clone M1/70, Material number 557397) for 30 min at 4 °C. Cells were washed with PBS twice. After the last wash, cells were resuspended with 100 µL PBS. Next, to detect intracellular NOS and CD206, an Intracellular Fixation and Permeabilization Buffer Set (Thermo Fisher Scientific; Catalog number: 88-8824) was used in accordance with the manufacturer’s instructions ^20^. Washed cells were fixed by adding 100 µL of Fixation Buffer and incubated for 30 min at room temperature in the dark. Then, 2 mL of Permeabilization Buffer was added and centrifuged at 500*g* for 5 min at room temperature. Cells were resuspend in 100 µL of Permeabilization Buffer and PE-conjugated rat anti-mouse NOS monoclonal antibody (Thermo Fisher Scientific; Clone CXNFT, Material number 25-5920-82) or FITC-conjugated rat anti-mouse CD206 monoclonal antibody (Thermo Fisher Scientific; Clone MR5D3, Material number MA5-16870) for the detection of intracellular antigens and incubated for 30 min at room temperature in the dark. After treatment with antibodies, 2 mL of Permeabilization Buffer was added and centrifuged at 500*g* for 5 min at room temperature. The supernatant was discarded, and cells were resuspended with 2 mL of Permeabilization Buffer and centrifuged at 500*g* for 5 min at room temperature. Then cells were suspended in PBS and analyzed by flow cytometry (Cytomics FC500, Beckman Coulter, Brea, CA, USA) for the direct detection of CD11b-positive/NOS-positive M1 macrophages and CD11b-positive/CD206-positive M2 macrophages^20^.

### Preparation of cultured BM stromal cells

For preparation of the stromal monolayer, BM cells were cultured at 1 × 10^6^ cells/mL in 6-well flat-bottom plates (Falcon 353046) in 4 mL per well of α-MEM supplemented with 10% fetal bovine serum. After three days of culture, the culture medium was removed completely, fresh culture medium was added, and the cells were cultured for another seven days. After 10 days of culture, the culture medium was removed completely and the cells were used for RNA extraction, β-galactosidase assays, and immunohistological analysis to detect the expression of Ki67 and phosphorylated ribosomal protein S6 (pRPS6)^21^.

### Gene expression assay

The levels of gene expression for cytokines were determined by real-time PCR using the Applied Biosystems 7500 Fast Sequence Detection System (Applied Biosystems, Foster City, CA, USA). Briefly, total RNA from BM cells and cultured stromal cells was isolated using ISOGEN reagent (Nippongene Corp., Toyama, Japan). mRNA was reverse transcribed using Superscript III (Life Technologies, Carlsbad, CA, USA) and oligo-dT (Promega Corp., Madison, WI, USA). The transcript levels were determined by real-time PCR using TaqMan™ Universal Fast PCR master mix (Applied Biosystems) and gene-specific primers. Specific primers and probes for the murine genes encoding granulocyte colony-stimulating factor (G-CSF), GM-CSF, interleukin (IL)-6, IL-7, stromal-cell derived factor-1 (SDF-1), stem cell factor (SCF), tumor necrosis factor-α (TNF-α), transforming growth factor-β (TGF-β), p16^INK4a^, and glyceraldehyde 3-phosphate dehydrogenase (GAPDH) were as described elsewhere^21–23^ and were purchased from Applied Biosystems.

### β-galactosidase assay

β-galactosidase activity was detected using the Cellular Senescence Kit (OZ Bioscience, San Diego, CA, USA) according to the manufacturer’s instructions. The percentage of cells positive for β-galactosidase was determined in duplicate in 500 cells per sample^21^.

### Immunohistological analysis for detecting the expression of Ki67 and pRPS6

BM stromal cells from young and aged SAMP1/TA-1 mice were washed with PBS, then fixed in a solution of 4% formaldehyde for 10 min. Cells were washed with PBS twice and permeabilized with 0.3% Triton-X100 (Sigma-Aldrich) on ice for 5 min and incubated in a blocking solution (5% fetal calf serum (FCS) solution in PBS and 0.1% Triton-X100) for 1 h at room temperature. Subsequently, samples were incubated with antibodies against pRPS6 (1:400, 4858, Cell Signaling Technology, Danvers, MA, USA) and Ki67 (718071, Nichirei Bioscience Inc., Tokyo, Japan) at 4 °C overnight. Then, we used a VECTASTAIN ABC Kit (PK-4001, VECTOR LABORATORIES, INC., Burlingame, CA, USA) and a HISTOFINE SAB-PO(M) Kit (425011, Nichirei Bioscience Inc.) as secondary antibodies to detect the expressions of pRPS6 and Ki67 on cultured stromal cells. The percentage of positive cells was calculated by the number of cells that expressed the specific marker stain out of at least 500 cells in different microscopic fields.

### Determination of the levels of GM-CSF and TNF-α produced by cultured BM stromal cells

Stromal monolayers were prepared by culturing bone marrow cells from young and aged SAMP1/TA-1 mice at 1 × 10^6^/mL in 24-well flat-bottomed plates (Falcon 353047) in 1 mL of IMDM supplemented with 10% FCS^42^. Confluent adherent layers were obtained after seven days. The supernatant in the culture plate was replaced with new culture medium consisting of 1 mL of IMDM supplemented with 10% FCS. The next day, 10 or 100 ng/mL of LPS was added to the culture plate, and the culture medium was collected after 48 h of culture and used to determine the levels of GM-CSF and TNF-α produced by stromal cells. In the control culture, PBS was added, and the culture medium was collected. The concentrations of GM-CSF and TNF-α in the culture medium were determined using GM-CSF and TNF-α-specific ELISA Kits (R&D Systems, Inc.) according to the manufacturer’s instructions. All samples were assayed in triplicate.

### Statistical analysis

Data are expressed as the mean ± standard deviation (SD). Data analyses between the non-treated group and LPS-treated group in young and aged SAMP1/TA-1 mice were performed using two-tailed unpaired Student’s *t* tests. Data analyses between young and aged SAMP1/TA-1 mice were performed using ANOVA with the Bonferroni test. Differences were considered statistically significant at P < 0.05.

## Data Availability

All data generated or analyzed during this study are included in this published article.
